# Insights from the protein sequence and structure analysis of PgHsc70 and OsHsp70 genes

**DOI:** 10.6026/97320630018088

**Published:** 2022-02-28

**Authors:** Gugulothu Baloji, Sandhya Jagtap, Ashwini Talakayala, Meghana Kolli, Lali Lingfa, Mallikarjuna Garladinne, Srinivas Ankanagari

**Affiliations:** 1 Plant Molecular Biology Laboratory, Agri Biotech Foundation, Rajendra Nagar, Hyderabad (T.S) 500 030, India; 2 Department of Genetics, Osmania University, Hyderabad - 50007 (T.S) India

**Keywords:** Abiotic stress, chaperones, Brassinolide, heat shock proteins, homeostasis, environmental stressors

## Abstract

Heat shock proteins are induced in a wide range of abiotic and biotic stresses. They are well known for cellular chaperone activities and play an important role in protecting plants through regulation of homeostasis and survival. A comprehensive characterization and comparative analysis of the Hsp70 family members within the closely related plant species helps in better interpretation of these proteins' contribution to cell function and response to specific environmental stresses. Therefore, it is of interest to glean insights from the protein sequence analysis of PgHsc 70 and OsHsp70 genes. Thus, we document data from the sequence and structure analysis of PgHsc 70 and OsHsp 70 gene a.

## Background:

Plant defense mechanisms are induced rapidly and plants adapt at morphological, molecular and physiological levels [1,2,3]. The sensing of abiotic stresses initiates complex signaling pathways controlling the stress and tolerance responses. The signal transmission of stress and subsequent induction of stress receptive pathways involves expression of genes and proteins related to tolerance that have been studied extensively at the molecular level [4,5]. The molecular mechanisms have identified a large number of genes induced with abiotic stress factors and characterized using approaches such as subtractive cDNA libraries [6], microarrays [7] and NGS based RNA sequencing [8]. In the cell, protein aggregation due to environmental stresses is a major effect resulting in their dysfunction. Understanding the protective mechanisms to abiotic stresses is indispensable for developing crops with increased stress tolerance [9].

In the environmental stress conditions, the cell survival and sustenance is dependent upon protein native conformation and preventing protein aggregation. In the event of environment stress conditions, chaperone proteins, assist to fold cellular proteins into three-dimensional conformation and avoid abnormal folding and aggregation [10,11]. Heat shock proteins (HSPs) are the central chaperone proteins, involved in the maintenance of homeostasis, nascent protein folding, denatured proteins refolding, aggregation prevention and aiding protein transport across the membranes [12,13]. Besides these processes, HSP gene family members also protects the cells from the damage caused in the events of extreme temperatures, salinity, dehydration, oxidative stress, heavy metal toxicity, high intense irradiation and wounding [14,7]. In the cells under higher temperature environments, the HSPs are immediately synthesized and expressed, and on the other hand, most of other proteins' synthesis is detained. Thus, heat shock proteins are performing key role in protecting plants through cellular homeostasis regulation to stress conditions [15,16]. Furthermore, based on stress signal, research has shown that high HSPs expression and accumulation is involved in different stress signaling pathways. In plants, the HSP induction, synthesis and increase in thermo tolerance are well documented [17,18,19].

Based on the molecular weights, five families of heat shock proteins are identified. The major HSPs families are chaperonin (Hsp60/GroEL), 70-kDa Hsp (Hsp70/DnaK), Hsp90, Hsp100/ClpB, and the small heat shock proteins (sHsp) [20]. Of these, the HSP70 family is of greatest interest. It is evolutionarily conserved, present in archaebacteria, plants and humans [21]. The four Hsp70 gene subgroup family members are localized in the sub-cellular compartments: plastids, mitochondria, endoplasmic reticulum and cytosol [22]. Furthermore, Hsp70 family includes genes that are constitutive (housekeeping) and predominantly associated with physiological functioning such as heat shock cognate (hsc) 70 gene, or stress-induced such as hsp70. In general, the newly synthesized proteins are folded by constitutive expressed members whereas protein translocation into the organelles, involve stress-induced members that re-fold and degrade mis-folded proteins in adverse environmental stress conditions [23,24]. Both these proteins have modular structure playing role in cell growth with conserved ATPase domain and hydrophobic pocket with lid-like structure of substrate-binding domain (SBD) at N-terminal end and variable C-terminal domain but conserved [23]. Because of diverse subcellular localizations, Hsp70 plays critical role in development or specific protein communications [25]. The activity of Hsp70 is also modulated by post-translational modifications and by interaction with other co-chaperones [26].

The yield improvement of crops in the unfavorable abiotic conditions is a challenge. In particular the role played by Poaceae crops in food demand is well known, contributing high amount of calories in the human diet [27,28]. Wheat, rice, maize, millet, sorghum, barley and rye starchy grains serving as important food sources for the world's majority population [27,28]. The Hsp70 family members comprehensive characterization in plant species is needed to know how these members contribute towards cell function and protect in adverse abiotic stresses 24]. Therefore, it is of interest to document the protein sequence analysis data of PgHsc 70 and OsHsp 70 genes to glean useful information.

## Materials and Methods:

### Data source:

The complete nucleotide sequence and the CDS of Pennisetum glaucum heat shock cognate 70 kDa protein (PgHSC70) and Oryza sativa hsp70 gene for heat shock protein 70 (OsHSP70) registered in GenBank and their protein sequences are obtained in FASTA format from National Center for Biotechnology information (NCBI). The gene and protein structural and functional analysis of sequences was done using in-silico tools. 

### Gene structure analysis:

The gene structure of PgHSC70 and OsHSP70 were predicted based on the genome and coding sequences using the Gene Structure Display Server. This server analyses exon/intron organization of PgHSC70 and OsHsp70 genes. In the gene structure analysis exons/introns and intronic phase distribution (phase 0, 1, 2) were identified and marked. Based on position relative to reading frame three intron phases exist: insertion between two codons (phase 0), insertion after first base codon (phase 1) or after second base codon (phase 2).

### Conserved motif analysis:

Using MEME suite sequence motifs were scanned over the nucleotide sequences in PgHSC70 and OsHSP70 genes. Input of cDNA sequences of PgHSC70 and OsHSP70 genes were given to the MEME suite. In a default setting, it can help in predicting up to three motifs and selected for finding distribution of motifs with three different parameters. Parameters were optimized in MEME suite set to 10 as maximum number and 1 as minimum occurrence of motif site per sequence. All the other parameters were kept in default value. Predicting the width and the occurrence number of motifs, in order to minimize the E-value, was automatically done with MEME suite.

### Cis-Acting regulatory elements:

Using Plant CARE database cis-acting regulatory elements (CREs) were scanned in PgHSC70 and OsHsp70 genes. The PgHSC70 and OsHsp70 genes cDNA sequences were uploaded and evaluated for cis-regulatory response elements presence in promoter regions to predict computationally the regulatory elements. For the response elements shown, a matrix value of ≥5 was considered for acceptance on the sense strand. The obtained cis-elements were compared with each other.

### miRNA Target sites prediction:

Plant small RNA-targeted gene prediction was performed on sequences PgHSC70 and OsHsp70 genes using psRNATarget server. The miRNA target sites were analyzed using default parameters.

### Multiple sequence alignment (MSA) and phylogenetic analysis:

For identification of similarity of PgHSC70 and OsHsp70, the homology search of each protein was performed by BLAST using blastp algorithm respectively. Using multiple sequence alignment (MSA) tool ClustalW2, the protein sequences of all the identified homologues of PgHSC70 and OsHsp70 were aligned. Using the BLOSUM 62 substitution matrix evolutionary alignment was inferred with progressive method. The phylogenetic tree was constructed for the identified PgHSC70 and OsHsp70 proteins using ClastalW2.

### Physicochemical characterization:

Physicochemical characterization of the target protein sequences of PgHSC70 and OsHsp70 such as mol. wt, aa composition, isoelectric point (pI), instability index (II), the total negative and positive residues, extinction coefficient (EC), grand average of hydropathicity (GRAVY) and aliphatic index (AI) were analyzed using Expasy's ProtParam prediction server.

### Secondary structure prediction:

SOPMA tool was used to predict PgHSC70 and OsHsp70 proteins secondary structure for assigning positional possibility of various regions of α-helix, β-strands, turns as well as random coils likely to fold. The method makes use of predicting consensus from multiple alignments of the relative frequencies of each amino acid anchored in the X-ray crystallographic solved protein templates.

### Prediction of Subcellular localization and SignalP:

PgHSC70 and OsHsp70 subcellular localization was predicted using CELLO v.2.5 which is a multiclass support vector machine classiﬁcation system. SignalP was used to verify the presence of signal peptide cleavage sites and their locations in both proteins, which works on the basis of a combination of several neural networks, namely artificial neural network (ANN) and Hidden Markov Model (HMM).

### Protein-Protein Interaction network:

Using STRING (Search Tool for Retrieval of Interacting Genes) v 9.1 protein-protein interactions (PPIs) analysis was done. The STRING repository consisted of PPIs concerning stable protein complexes, functional and regulatory interactions. The PPIs of PgHSC70 and OsHsp70 were searched individually by submitting a protein query sequence in the search box of STRING. Determining the protein–protein interaction network would empower study of signaling pathways.

### Disulﬁde-Bonding in protein:

In protein folding and formation a functional and stable confirmation is determined by disulfide bonds among its cysteine residues. To predict cysteine bonds (disulfide bonds) presence and absence and their bonding patterns CYS_REC tool was used.

### Post-translational modification sites:

The targets in PgHSC70 and OsHsp70 were predicted for putative acetylation, methylation, phosphorylation, ubiquitination, and N-glycosylation sites. phosphorylation at serine, threonine and tyrosine residues in the PgHSC70 and OsHsp70 proteins were predicted using NetPhos 3.1 was used. To complete this task it ensembles neural networks and residues having scores 0.5 threshold as phosphorylated. The N-glycosylation sites of the target proteins were predicted with NetNglyc 1.0 server, with threshold value of >0.5. By default the predictions are done only on the Asn-Xaa-Ser/Thr sequons.

### Protein disorder analysis:

The estimation of intrinsic disordered regions (IDRs) of PgHSC70 and OsHsp70 was made by DisEMBL tool.

### Homology modeling and docking:

The protein structures of PgHSC70 and OsHsp70 was modeled using the bovine HSC70 (PDB ID: 1YUW) as template sequence exhibiting the highest similarity identified by BLAST against the PDB database. DS was used to design a homology model of both proteins and the each protein model with less geometric function was selected and energy minimized in CHARMm force field using DS minimization algorithms. For structural validation, the obtained final models are further subjected to PROCHECK for Psi/Phi Ramachandran plots analysis. Protein binding/catalytic sites are identified using DS Analyze Binding Site tool.

### Molecular docking:

The homology models of PgHSC70 and OsHSP70 were analyzed for docking with brassinolide. Using DS LibDock docking simulation was performed. The obtained confirmations were then summarized and analyzed for interactions. The interactions showing highest scores and docking energy were considered best for protein-ligand complex structure.

## Results and Discussion:

Comparative alignment of genomic and cDNA nucleotide sequences of PgHsc70 and OsHSP70 genes is shown in Figure 1(see PDF). The intron phases consisted of phase 0, 1 and 2. Exon count and intronic phase distribution was similar in both the genes. Two exons were present in both the genes and their distribution was found at phase 0 (exon 1) and phase 2 (exon 2). Phase 1 intronic phase distribution was found in both the genes. The intron length varied with OsHSP70 intron of 1935 bp while PgHSC70 intron is 141 bp. The MEME tool identified 10 significant conserved motifs as shown in Figure 2(see PDF). The length of conserved motifs varied from 21 to 50 amino acids. The consensus sequence motifs identified are given in Table 1(see PDF).

In silico analysis of cis-regulatory elements (CREs) in the CDS sequence of PgHSC70 and OsHsp70 genes revealed different elements in the upstream region (Table 2-see PDF). In PgHSC70 a total of 33 CAREs whereas, in OsHSP70, 29 CAREs were identified and a few were uniquely present in each gene. Cis-elements were found responsive to light, meristem-specific activation, abscisic acid, methyl jasmonate, gibberellins, low temperature, seed-specific regulation, root-specific expression, anoxia, and circadian regulation. The CCGTCC motif, ABRE, W box, CCAAT-box, GC motif,, G-Box, MYB recognition site STRE, MYB, TGA-element, plant_AP-2-like, WRE3, and unnamed-1 are the common CREs found in both the genes. All of them were present on sense strand with matrix value ≥ 5.

The plant small RNA target analysis server (psRNATarget) was used to predict miRNA target sites. The miRNAs comprising target sites in PgHSC70 and OsHSP70 genes were identified with expectation score lower than 4.0 (Table 3 -see PDF)). MSA using ClustalW was constructed by aligning seven PgHSC70 protein sequences along with twelve OsHsp70 protein sequences. Phylogenetic tree based on MSA is shown in Figure 3(see PDF). Comparative phylogenetic analysis of PgHSC70 and OsHsp70 revealed major groups of HSP70 genes with paralogous as well as orthologous genes. Each group contained both PgHSC70 and OsHsp70.

The Expasy's ProtParam tool determined the length (649 and 648 aa), mol. wt. (71105.48 and 70997.56 Da), isoelectric point (pI=5.10 and 5.16), the total negative and positive charged residues of PgHSC70 and OsHsp70 proteins (100 and 98 and 82 and 82, respectively). Proteins are found be acidic (pI<7). The amino acid composition analysis revealed that Ala (58) was the most abundant amino acid in both the sequences, while Trp (3) was the least abundant (Table 4-see PDF). PgHsc 70 had the atomic composition C2777H4999N861O995S24, while OsHsp70 had the atomic composition C3108H5007N861O987S24.

The calculated extinction coefficient (EC) of PgHsc70 and OsHsp70 proteins was found to correlate with the cysteine, tryptophan and tyrosine content at 280 nm as 37735 and 39225 (assuming all pairs of cysteine residues form cysteines) and 37360 and 38850 (assuming all cysteine residues are reduced) M−1cm−1, respectively. The instability index (II) values were 34.52(PgHsc70) and 33.69(OsHsp70) classifying both proteins as stable. Similarly, the aliphatic index (AI) had high value of 81.79 (PgHsc70) and 83.56 (OsHsp70), indicating proteins are thermo stable.

The estimated half-lives for both proteins were 30 h in mammalian reticulocytes (in vitro), >20 h in yeast (in vivo) and >10 h in Escherichia coli (in vivo). The GRAVY was -0.427 (PgHsc70) and -0.399 (OsHsp70), respectively. Both proteins are highly water soluble. Table 5(see PDF) shows the predicted physicochemical properties of the PgHSC70 and OsHsp70. In Figures 4 and 5(see PDF), the secondary structure of protein sequences of PgHSC70 and OsHsp70, predicted using SOPMA server are shown. The evaluated percentage α-helices, β-turn, extended strand, and random coils with output width 70 is given in Table 6(see PDF). From the computed percentage of each conformation, α-helix predominated, followed by extended strand random coil and random coil in both the proteins. The high percentage of random coils indicates protein flexibility and more interactions. Also, high coiled structural content might be because of flexible glycine and proline amino acids in the proteins.

The sub cellular localization of proteins PgHsc70 and OsHsp70 predicted by CELLO was found to be cytosolic in nature. The SignalP analysis revealed that none of the proteins have any of signal peptide. Using CYS_REC tool the cysteine residues in the proteins determined revealed that the protein PgHsc70 contain cysteine residues in the positions 20, 273, 319, 326, 366, 483 and 609. But all are not involved in disulﬁde bonding; Cys326 is probably SS-bond with a score of 1.9. On the other hand, OsHsp70 revealed that cysteine residues were present in the position 20, 272, 318, 325, 365, 482 and 608 and probably Cys326 is SS-bonded with a score of 1.9. At the molecular level the disulfide bridges presence is a positive factor for stability. Table 7(see PDF) shows the results obtained using CYS_REC tool.

NetPhos 3.1 & NetNglyc servers identify post-translational modification sites. NetPhos retrieved the information related to kinases PKC, Unsp, CKI, cdk5, CKII, PKA, RSK, DNAPK, CKI, PKG, EGFR , SRC and cdc2 involved in the phosphorylation of both proteins and an extra one kinase INSR unique to OsHsp70. In both the proteins high score of 0.691 predicted for site having threonine and the percentage amino acids composition obtained is shown in Figures 6a and 6b(see PDF). Using NetNglyc server, the N-glycosylation sites (38 NRTT, 155 NDSQ, 423 NTTI and 493 NVSA) of the PgHsc70 protein were predicted with a score of 0.66, 0.57, 0.58 and 0.74 respectively. The N-glycosylation sites of OsHsp70 protein predicted were (38 NRTT, 154 NDSQ, 422 NTTI and 492 NVSA) with scores of 0.6378, 0.5774, 0.5874 and 0.7403. The plot of predicted N-glycosylation sites is shown in Figures 7a and 7b(see PDF).

Using String, the interacting partners predicted in both PgHsc70 and OsHsp70 is shown in the Figure 8(see PDF). From the analysis, the functional partners observed in the string network of PgHsc70 protein were HSFA2, HSF1, HSP101, HSP90.1, Hsp81.4, Hop3, HSP81-3, LOS1, J3 and Hop1. The functional partners observed in the string network of OsHsp70 are OsJ-17347, OS11T0703900-01, OsJ_11911, HSP81-2, OsJ_12871, DJA6, and OS04T0107900-02. These interactions give some insights into understanding the functioning of these proteins in response to heat stress and tolerance. Using DisEMBL the predicted intrinsic disorder regions (IDRs) of PgHSC70 and OsHsp70 is shown in Figure 9(see PDF).

Homology modeling was done to predict the 3-D structures of PgHSC70 and OsHsp70 based on the template structure HSC70 (PDB ID: 1YUW) from bovine, at a resolution of 2.6 Å deposited in PDB. The template protein had identity of 81% (PgHSC70) and 80.18 % (OsHsp70). The initial models of PgHSC70 and OsHsp70 proteins were built using the crystal coordinates information of the template 1YUW. The models generated by DS, were scored with discrete optimized protein energy (DOPE) geometric function, and the model with the lowest DOPE score was taken as final model as shown in Figure 10(see PDF). After the proteins were energy minimized, the final models were validated using PROCHECK. Corresponding to core regions most favorable Psi/Phi value combinations are present in the darkest areas in Ramachandran plot. Each of the protein models displayed 90% accuracy. Overall, the homology model of the PgHSC70 have 94.6% of the residues occurring in most favored region, 4.4 % in allowed regions, and only 1.1 % of the residues in disallowed regions. In comparison, the OsHsp70 homology model have 94.6% residues in favored region, 4.9 % residues in allowed region and 0.5 % residues in outlier region.

Molecular docking of PgHSC70 and OsHsp70 with brassinolide was studied in order to identify the critical interactions and their variation. Using LibDock the docking results for brassinolide on PgHSC70, showed high binding affinity with score of 115.231 and binding energy of 0.00119 kcal/mol, in comparison target OsHsp70 showed a LibDock score of 146.59 and binding energy of -26.586 kcal/mol. In the PgHSC70-Brassinolide complex, the electrostatic and −87.3 and -835.7 kcal/mol of van der Waals energies respectively and for OsHsp70-Brassinolide complex -18.332 and 4.139 kcal/mol were found to be higher. Docking analysis revealed both H-bonds and close interactions within the docked site of PgHSC70 and OsHsp70 (Figure 11-see PDF). The PgHSC70-Brassinolide complex formed five hydrogen bonds, 3 with residue THR271, each one with LYS59 and LYS277, and the closest interactions are also found with the amino acid residue GLY236. Whereas the OsHsp70-Brassinolide complex formed two hydrogen bonds with two residues ASP35 and LYS129 and found to interact with the amino acid residue ILE133. The docking studies clearly indicated that the ligand and receptor were bound together closely to stabilize complex structure in OsHsp70 and PgHsc 70 as done in our previous study [29].

## Conclusion:

We have documented the characterization of Hsp70 gene family members, PgHSC70 and OSHsp70 genes, and their proteins sequences in pearl millet and rice respectively. The results indicated conserved relationships and distinct functions of PgHSC70 and OSHsp70 highlighting the wide participation of these family members in environmental adaptation. Data from docking analysis of the homology models with brassinolide is also reported.
